# A giant colonic diverticulum presenting as a 'phantom mass': a case report

**DOI:** 10.1186/1752-1947-3-29

**Published:** 2009-01-27

**Authors:** Ayman S Abdelrazeq, Anwar E Owais, Munther I Aldoori, Ian D Botterill

**Affiliations:** 1 North Cheshire NHS Trust Hospitals, Lovely Lane, Warrington, WA5 1QG, UK; 2 Department of General Surgery, Scarborough General Hospital, Scarborough, UK; 3 General surgery, Huddersfield Royal Infirmary, Huddersfield, UK; 4 Department of Colorectal Surgery, St. James's University Hospital, Leeds, UK

## Abstract

**Introduction:**

Diverticulosis coli is the most common disease of the colon in Western countries. Giant colonic diverticulum, defined as a colonic diverticulum measuring 4 cm in size or larger, represents an unusual manifestation of this common clinical entity.

**Case presentation:**

A 68-year-old Caucasian British woman with a history of intermittent lower abdominal mass, leg swelling and focal neurological symptoms underwent extensive non-diagnostic investigations over a significant period under a number of disciplines. The reason for a diagnosis being elusive in part related to the fact that the mass was never found on clinical and ultrasound examination. As a result, the patient's validity was questioned. Ultimately, this 'phantom-mass' was diagnosed as a giant colonic diverticulum causing intermittent compression of the iliac vein and obturator nerve.

**Conclusion:**

Intermittent compression of the iliac vein and the obturator nerve by a colonic diverticulum has not previously been reported. A giant colonic diverticulum presenting as an intermittent mass is very rare. This case also illustrates two factors. First, the patient is often right. Second, the optimal mode of investigation for any proven or described abdominal mass with referred symptoms is cross-sectional imaging, typically a computed tomography scan, irrespective whether the mass or symptoms are constant or intermittent.

## Introduction

Giant colonic diverticulum (GCD) defined as a colonic diverticulum measuring 4 cm in size or larger is a rare disease [1]. It is nearly always associated with diverticular disease.

Several theories have been proposed to explain its aetiology: a unidirectional flap-valve mechanism at the base of the diverticulum allowing bowel gas and debris to enter but not to leave the diverticulum; gas-forming organisms distend a diverticulum after its mouth has become obliterated; an organized abscess that develops around an infected pre-existing pseudodiverticulum; or a true congenital duplication [1,2]. The exact aetiology remains unknown.

Here we report a patient with GCD who presented with some peculiarities, which we believe will be valuable in further explaining the mechanism behind GCD, and may help in the prompt diagnosis and treatment of future similar cases.

## Case presentation

A 68-year-old Caucasian British woman presented with a history of an intermittent large painless mass in her lower abdomen. The mass was more pronounced before defecation and was associated with paraesthesia in the medial side of the left thigh and swelling of the left lower limb. Following defecation, the mass disappeared with instant relief of the associated symptoms. She had no other associated complaints and clinical examination was normal.

Blood tests and abdominal ultrasound were normal. Colonoscopy showed sigmoid diverticulosis. She was reassured and discharged.

The patient's symptoms recurred and she was referred for a second opinion from a gastroenterologist, a urologist, and a neurologist. Clinical and neurological examination was again confirmed to be normal. An upper gastrointestinal series, intravenous pyelogram, cystoscopy, MRI spine and venous duplex scan were all reported as unremarkable, though at the time of these tests, the patient was asymptomatic. The patient was referred to a psychiatrist and subsequently commenced on various sedatives and antidepressants with no improvement.

**Figure 1:**
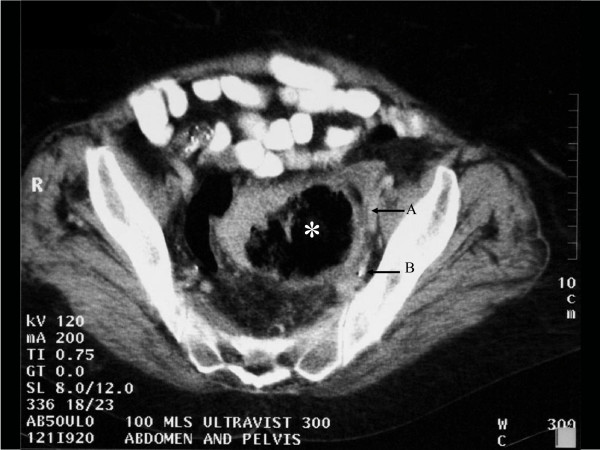
Axial computed tomography image with oral and intravenous contrast at the level of S1 demonstrating a giant colonic diverticulum*. The wall of the diverticulum and the surrounding fat are thickened denoting recent inflammation. Note the relationship to the left external and internal iliac vessels (arrows A and B, respectively). The obturator nerve (L2–4) emerges from the medial aspect of the psoas muscle and runs downwards and forwards, deep into [the internal iliac vessels.

Two years later, she was referred to our team with similar ongoing complaints. Clinical examination remained unremarkable. Barium enema confirmed uncomplicated diverticular disease. Shortly following barium enema she developed severe lower abdominal pain and fever. Computerized tomography demonstrated a thick-walled, air filled cavity closely related to the sigmoid colon, compatible with a GCD (Figure 1).

At laparotomy, an 8 cm mass was found to originate from the antimesenteric border of the sigmoid colon. It was adherent to the iliac vessels and lateral pelvic wall. Diverticulosis involving mainly the sigmoid colon was noted. Sigmoid colectomy was performed. She had an uncomplicated recovery. At 12-month follow-up, she remained asymptomatic.

Pathology confirmed a GCD with diverticulitis and peritonitis. This diverticulum was 8 cm across with a 5 mm luminal orifice and was filled with air and faecal debris; a flap-valve effect was noted at the opening of the colon. [Uninflamed simple diverticula were also noted.

## Discussion

Diverticulosis coli is the most common disease of the colon in Western countries; GCD represents an unusual manifestation of this common clinical entity [3].

Histologically, there are three different types of GCD with similar clinical presentations [2]. The first type is a pseudo-diverticulum which gradually increases in size; remnants of muscularis mucosa and muscularis propria may be found in its wall. In most cases, the mucosa is not completely intact. This type of GCD could be explained by the flap-valve or gas-forming organisms theories.

If no mucosal remnants are found, the GCD is considered inflammatory (type 2), which is actually a result of a local perforation of the mucosa with an abscess cavity that remains in contact with the lumen of the colon (organized abscess theory). The wall of this diverticulum contains reactive scar tissue only. The third type is a true diverticulum, in which the wall contains all of the layers of normal bowel (congenital duplication theory).

In this patient, the diverticulum gradually inflated because of a flap-valve action of the tiny opening from the bowel, allowing gas and debris to enter, but not escape, during periods of straining. The failure of barium to enter the GCD attests to the small size of the opening. The fact that this GCD could expand and shrink indicates that its wall had elastic properties. When fully expanded, it produced compressive symptoms.

Symptoms of GCD and their duration are variable; often the patient's complaint can be ascribed to the associated diverticulitis [4]. A palpable abdominal mass in a patient with diverticulitis is almost invariably a paracolic inflammatory mass related to acute diverticulitis. Presentation of colonic diverticula as a palpable abdominal mass in the absence of acute diverticulitis is extremely rare [5]. The intermittent appearance of a large painless mass in our patient was probably due to filling of the GCD with gas and faecal material whose expulsion led to the disappearance of the mass. The associated symptoms are related to pressure on the left iliac vein and the left obturator nerve.

Such a presentation of GCD as a longstanding non-inflammatory intermittent abdominal mass is distinctly unusual. Intermittent compression of the iliac vein or the obturator nerve as a presentation of GCD has not previously been reported.

Plain film diagnosis of GCD can be made in the presence of a persistent smooth-walled gas containing structure, 'balloon sign', adjacent to the colon with or without air fluid level [6].

Diagnostic colonoscopy is not considered to be helpful [7] except in cases with a large ostium where an incidental diagnosis of GCD is possible [8]. The use of barium enema did not yield a positive diagnosis in this patient, although a communication with the colon can be demonstrated on contrast studies in 25% to 66% of GCD cases [7].

Ultrasound examination does not seem to be helpful in detecting a non-complicated GCD [7]. In our patient, we were falsely reassured by a normal ultrasound. Computerized tomography and magnetic resonance imaging are useful in defining the GCD and its relation to surrounding structures [9]. The intermittent nature of this patient's symptoms made diagnosis difficult. Arguably, an earlier CT scan may have helped. The final diagnosis was only made when barium inspissated in the neck of the GCD and provoked local diverticulitis which merited immediate CT evaluation.

## Conclusion

This case of GCD which presented initially with a longstanding non-inflammatory intermittent abdominal mass is distinctly unusual. Intermittent compression of the iliac vein or the obturator nerve as a presentation of GCD has not previously been reported.

This case also illustrates two factors. First, the patient is often right. Second, a proven or described abdominal mass with or without referred symptoms should prompt cross-sectional imaging, typically a CT scan, irrespective of whether the mass or symptoms are constant or intermittent.

## Abbreviations

CT: computed tomography; GCD: giant colonic diverticulum; MRI: magnetic resonance imaging

## Consent

Written informed consent was obtained from the patient for publication of this case report and any accompanying images. A copy of the written consent is available for review by the Editor-in-Chief of this journal.

## Competing interests

The authors declare that they have no competing interests.

## Authors' contributions

AA and AO identified the association between the GCD and the patient's unique presentation, reviewed the literature and drafted the manuscript. MA and IB contributed to drafting the manuscript and critically revised the discussion and conclusions. All authors have read and approved the manuscript.
